# Non-contiguous finished genome sequence of *Corynebacterium timonense* type strain 5401744^T^

**DOI:** 10.4056/sigs.4277954

**Published:** 2014-04-25

**Authors:** Véronique Roux, Catherine Robert, Didier Raoult

**Affiliations:** 1 Aix Marseille Université, Faculté de médecine, Aix-Marseille Université, France

**Keywords:** *Corynebacterium timonense*, *Actinobacteria*

## Abstract

*Corynebacterium timonense* strain 5401744^T^ is a member of the genus *Corynebacterium* which contains Gram-positive bacteria with a high G+C content. It was isolated from the blood of a patient with endocarditis. In this work, we describe a set of features of this organism, together with the complete genome sequence and annotation. The 2,553,575 bp long genome contains 2,401 protein-coding genes and 55 RNA genes, including between 5 and 6 rRNA operons.

## Introduction

*Corynebacterium timonense* strain 5401744^T^(CSUR P20^T^=CIP 109424^T^= CCUG 53856^T^) is the type strain of *C. timonense.* This bacterium was isolated from the blood of a patient with endocarditis [1]. The genus *Corynebacterium* is comprised of Gram-positive facultatively anaerobic bacteria with a high G+C content. It currently contains over 80 members [2]. The combination of chemotaxonomic markers [3,4] and a molecular approach based on 16S rRNA and *rpoB* gene sequence analyses improved the identification of members of this genus [5-7]. *Corynebacterium* species have been isolated from human clinical sources [8-14], animal sources [15-18] and the environment [19-21].

Here we present a summary classification and a set of features for *C. timonense,* together with the description of the non-contiguous finished genomic sequencing and annotation.

## Classification and features

**Figure 1:**
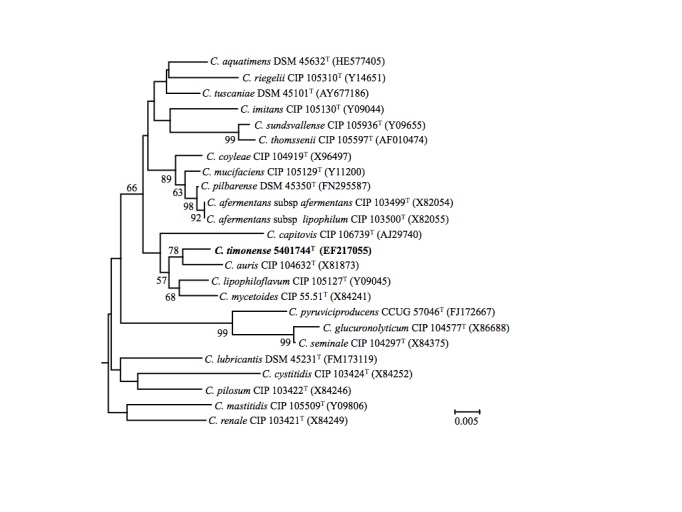
Part of phylogenetic tree highlighting the position of *Corynebacterium timonense* strain 5401744^T^ relative to other type strains within the *Corynebacterium* genus by comparison of 16S rRNA gene sequences. GenBank accession numbers are indicated in parentheses. Sequences were aligned using CLUSTALX, and phylogenetic inferences obtained using the neighbor joining method within the MEGA 5 software [22]. Numbers at the nodes are percentages of bootstrap values (≥ 50%) obtained by repeating the analysis 1,000 times to generate a majority consensus tree. *Solibacillus silvestris* was used as outgroup. The scale bar represents 0.005 nucleotide change per nucleotide position.

The 16S rRNA gene sequence of *C. timonense* strain 5401744^T^ was compared with sequences deposited in the Genbank database, confirming the initial taxonomic classification. Figure 1 shows the phylogenetic neighborhood of *C. timonense* in a 16S rRNA based tree. The bacterium was first characterized in July 2005, in a 56-year-old man with a history of infective endocarditis. It was isolated from blood culture in the Timone Hospital microbiology laboratory.

**Figure 2:**
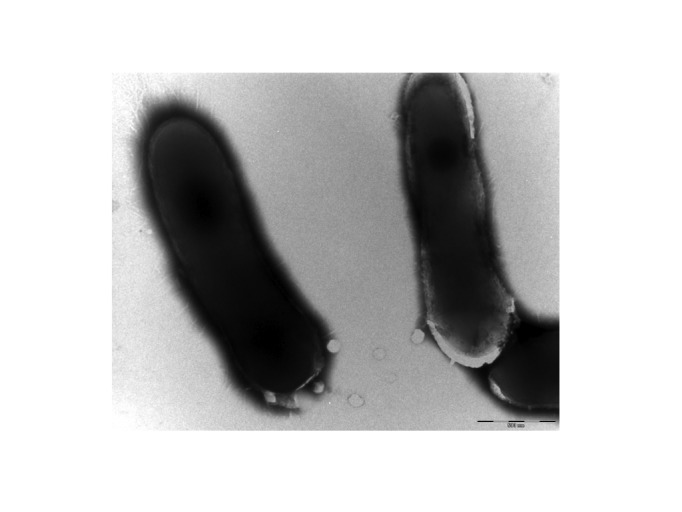
Transmission electron micrograph of *C. timonense* strain 5401744^T^, using a Morgani 268D (Philips) at an operating voltage of 60kV. The scale bar represents 500 nm.

Cells are rod-shaped that occur as single cells, in pairs or in small clusters, 0.6-2.1 µm long and 0.4-0.6 µm wide. Optimal growth of strain 5401744^T^ occurs at 37°C with range for growth between 25 and 50 °C. After 24 hours growth on blood sheep agar at 37°C, surface colonies are circular, yellow colored, glistening and up to 1-2 mm in diameter. Carbon sources utilized include glucose and ribose. Activities of catalase, pyrazinamidase, alkaline phosphatase, esterase (C4), esterase lipase (C8), lipase (C14), leucine arylamidase and acid phosphatase are detected. The fatty acid profile is characterized by the predominance of C18:1 ω9c (36.4%), C17:1 ω9c (27.1%), C16:0 (10.9%) and C18:0 (6.1%). Tuberculostearic acid is not detected. The size and ultrastructure of cells were determined by negative staining transmission electron microscopy. The rods were 0.6-2.1 μm long and 0.4-0.6 μm wide (Figure 2). Table 1 presents the classification and features of the organism.

**Table 1:** Classification and general features of *Corynebacterium timonense* strain 5501744^T^

**MIGS ID**	**Property**	**Term**	**Evidence code^a^**
		Domain *Bacteria*	TAS [23]
		Phylum *Actinobacteria*	TAS [24]
		Class *Actinobacteria*	TAS [25]
	Current classification	Order *Actinomycetales*	TAS [25-28]
		Family *Corynebacteriaceae*	TAS [25,26,28,29]
		Genus *Corynebacterium*	TAS [26,30,31]
		Species *Corynebacterium timonense*	TAS [1]
		Strain 5401744^T^	TAS [1]
	Gram stain	Positive	IDA
	Cell shape	Pleomorphic forms	IDA
	Motility	Non-motile	IDA
	Sporulation	Non-sporulating	IDA
	Temperature range	Mesophile	IDA
	Optimum temperature	37°C	IDA
MIGS-6.3	Salinity	Not reported	IDA
MIGS-22	Oxygen requirement	Aerobic and facultatively anaerobic	IDA
	Carbon source	Glucose, ribose	NAS
	Energy source	Chemoorganotroph	NAS
MIGS-6	Habitat	Host	IDA
MIGS-15	Biotic relationship	Free living	IDA
MIGS-14	Pathogenicity Biosafety level Isolation	Unknown 2 Human blood sample	NAS
MIGS-4	Geographic location	Marseille, France	IDA
MIGS-5	Sample collection time	July 2005	IDA
MIGS-4.1	Latitude	43°18 N	IDA
MIGS-4.1	Longitude	5°23 E	IDA
MIGS-4.3	Depth	Surface	IDA
MIGS-4.4	Altitude	21 m above sea level	IDA

Evidence codes - IDA: Inferred from Direct Assay; TAS: Traceable Author Statement (i.e., a direct report exists in the literature); NAS: Non-traceable Author Statement (i.e., not directly observed for the living, isolated sample, but based on a generally accepted property for the species, or anecdotal evidence). These evidence codes are from the Gene Ontology project [32]. If the evidence is IDA, then the property was directly observed for a live isolate by one of the authors or an expert mentioned in the acknowledgements.

## Genome sequencing and annotation

### Genome project history

The organism was selected for sequencing on the basis of its phylogenetic position and 16S rRNA similarity to other members of the genus *Corynebacterium*, and is part of a study of the new species characterized in our laboratory. A summary of the project information is shown in Table 2. The EMBL accession number is CAJP01000000 and consists of 58 contigs (≥ 500 bp) and 10 scaffolds (> 4,375 bp). Table 2 shows the project information and its association with MIGS version 2.0 compliance.

**Table 2:** Project information

**MIGS ID**	**Property**	**Term**
MIGS-31	Finishing quality	High-quality draft
MIGS-28	Libraries used	One paired end 3-kb library and one Shotgun library
MIGS-29	Sequencing platforms	454 GS FLX Titanium
MIGS-31.2	Fold coverage	37.2×
MIGS-30	Assemblers	Newbler version 2.5.3
MIGS-32	Gene calling method	Prodigal
	EMBL ID	CAJP01000000
	EMBL Date of Release	February, 2, 2013
	Project relevance	Study of new species isolated in the URMITE

### Growth conditions and DNA isolation

*C. timonense* strain 5401744^T^, was grown aerobically on 5% sheep blood-enriched Columbia agar at 37°C. Five petri dishes were spread and colonies scraped and resuspended in 3 ml of TE buffer. Three hundred μl of 10% SDS and 150 μl of proteinase K were then added and incubation was performed over-night at 56°C. The DNA was then extracted using the phenol/chloroform method. The yield and the concentration was measured by the Quant-it Picogreen kit (Invitrogen) on the Genios Tecan fluorometer at 182 ng/µl.

### Genome sequencing and assembly

Shotgun and 3-kb paired-end sequencing strategies were performed. The shotgun library was constructed with 500 ng of DNA with the GS Rapid library Prep kit (Roche). For the paired-end sequencing, 5 µg of DNA was mechanically fragmented on a Hydroshear device (Digilab) with an enrichment size at 3-4 kb. The DNA fragmentation was visualized using the 2100 BioAnalyzer (Agilent) on a DNA labchip 7500 with an optimal size of 3.5 kb. The library was constructed according to the 454 GS FLX Titanium paired-end protocol. Circularization and nebulization were performed and generated a pattern with an optimal size of 501 bp. After PCR amplification through 15 cycles followed by double size selection, the single stranded paired-end library was then quantified using the Genios fluorometer (Tecan) at 2,540 pg/µL. The library concentration equivalence was calculated as 9.30E+09 molecules/µL. The library was stored at -20°C until further use.

The shotgun and paired-end libraries were clonally-amplified with 2 cpb and 1 cpb in 3 SV-emPCR reactions with the GS Titanium SV emPCR Kit (Lib-L) v2 (Roche). The yields of the emPCR were 11.5% and 7.92%, respectively, in the 5 to 20% range from the Roche procedure. Approximately 790,000 beads for the shotgun application and for the 3kb paired end were loaded on the GS Titanium PicoTiterPlate PTP Kit 70x75 and sequenced with the GS FLX Titanium Sequencing Kit XLR70 (Roche). The run was performed overnight and then analyzed on the cluster through the gsRunBrowser and Newbler assembler (Roche). A total of 252,118 passed filter wells were obtained and generated 37.19 Mb with a length average of 366.5 bp. The passed filter sequences were assembled using Newbler with 90% identity and 40 bp as overlap. The final assembly identified 10 scaffolds and 46 large contigs (>1,500 bp).

### Genome annotation

Open Reading Frames (ORFs) were predicted using Prodigal [33] with default parameters but the predicted ORFs were excluded if they spanned a sequencing GAP region. The predicted bacterial protein sequences were searched against the GenBank database [34] and the Clusters of Orthologous Groups (COG) database [35] using BLASTP. The tRNAscan-SE tool [36] was used to find tRNA genes, whereas ribosomal RNAs were found by using RNAmmer [37].

Transmembrane domains and signal peptides were predicted using TMHMM [38] and SignalP [39], respectively. ORFans were identified if their BLASTp *E-*value was lower than 1e-03 for alignment length greater than 80 amino acids. If alignment lengths were smaller than 80 amino acids, we used an *E*-value of 1e-05. Such parameter thresholds have been used in previous works to define ORFans.

To estimate the mean level of nucleotide sequence similarity at the genome level between *C. timonense* and the corynebacterium genomes available to date, we compared the ORFs only using comparison sequence based in the server RAST [40] at a query coverage of ≥60% and a minimum nucleotide length of 100 bp.

## Genome properties

**Figure 3:**
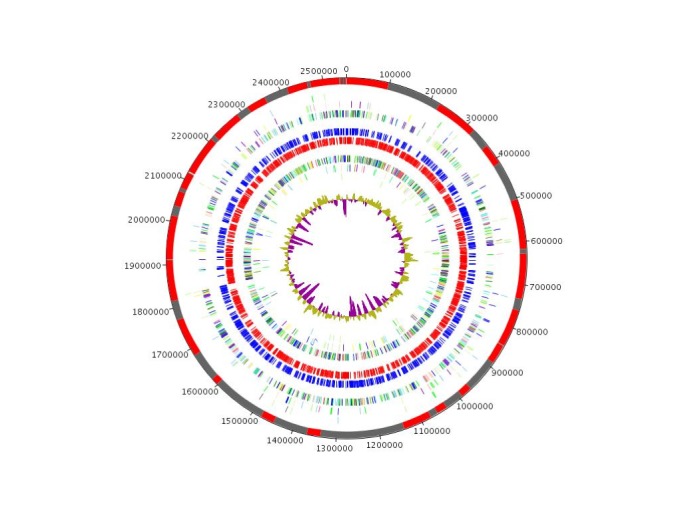
Graphical circular map of *Corynebacterium timonense* genome. From outside to the center: Contigs (red / grey), COG category of genes on the forward strand (three circles), genes on forward strand (blue circle), genes on the reverse strand (red circle), COG category on the reverse strand (three circles), GC content.

The genome is 2,553,575 bp long with a 66.85% GC content (Table 3, Figure 3). Of the 2,456 predicted genes, 2,401 were protein-coding genes, and 55 were RNAs. A total of 1,779 genes (74.09%) were assigned a putative function,and 116 genes were identified as ORFans (4,83%). The remaining genes were annotated as hypothetical proteins (369 genes (15,37%)). The remaining genes were annotated as either hypothetical proteins or proteins of unknown functions. The distribution of genes into COGs functional categories is presented in Table 4. The properties and the statistics of the genome are summarized in Tables 3 and 4.

**Table 3:** Nucleotide content and gene count levels of the genome

Attribute	Value	% of total^a^
Genome size (bp)	2,553,575	100
DNA coding region (bp)	2,289,384	89.65
DNA G+C content (bp)	1,707,056	66.85
Total genes	2,456	100
RNA genes	55	2.24
Protein-coding genes	2,401	97.76
Genes with function prediction	1,779	74.09
Genes assigned to COGs	1,753	73.01
Genes with peptide signals	353	14.7
Genes with transmembrane helices	550	22.91

a) The total is based on either the size of the genome in base pairs or the total number of protein coding genes in the annotated genome.

**Table 4:** Number of genes associated with the 25 general COG functional categories

**Code**	**Value**	**%age**	**Description**
J	148	6.16	Translation
A	1	0.04	RNA processing and modification
K	136	5.66	Transcription
L	179	7.46	Replication, recombination and repair
B	0	0	Chromatin structure and dynamics
D	17	0.71	Cell cycle control, mitosis and meiosis
Y	0	0	Nuclear structure
V	45	1.87	Defense mechanisms
T	62	2.58	Signal transduction mechanisms
M	89	3.71	Cell wall/membrane biogenesis
N	2	0.08	Cell motility
Z	0	0	Cytoskeleton
W	0	0	Extracellular structures
U	27	1.12	Intracellular trafficking and secretion
O	60	2.50	Posttranslational modification, protein turnover, chaperones
C	97	4.04	Energy production and conversion
G	121	5.04	Carbohydrate transport and metabolism
E	205	8.54	Amino acid transport and metabolism
F	65	2.71	Nucleotide transport and metabolism
H	100	4.16	Coenzyme transport and metabolism
I	78	3.25	Lipid transport and metabolism
P	176	7.33	Inorganic ion transport and metabolism
Q	46	1.92	Secondary metabolites biosynthesis, transport and catabolism
R	233	9.7	General function prediction only
S	137	5.71	Function unknown
X	648	26.99	Not in COGs

The total is based on the total number of protein coding genes in the annotated genome.

## Comparison with other *Corynebacterium* genomes

To date, 13 genome of species belonging to the genus *Corynebacterium* were sequenced. The size of the whole genome was between 2.32 Mb and 3.43 Mb (Table 5). The gene number was correlated with the genome size and was between 2,187 and 3,131. The G+C content of the genome was less than 60% for *C. diphteriae*, *C. glutamicum*, *C. kroppenstedtii*, *C. pseudotuberculosis*, *C. resistens* and *C. ulcerans* but was more than 60% for *C. aurimucosum*, *C. efficiens*, *C. genitalium*, *C. halotolerans*, *C. jeikeium*, *C. timonense*, *C. urealyticum* and *C. variabile*. *C. timonense* shared a mean sequence similarity of 72.05% (60-99.01%), 72.15% (60.09-97.54%), 74.63% (60-98.37%), 71.83% (60-98.85%), 72.34% (60-98.02%) and 71.70% (60-97.03%) with *C. diphteriae*, *C. efficiens*, *C. genitalium*, *C. glutamicum*, *C. jeikeium* and *C. urealyticum*, respectively.

**Table 5:** Comparison of *C. timonense* characteristics with *Corynebacterium* whole genome characteristics.

**Species**	**Genome size (Mb)**	**G+C%**	**Number of predicted genes**
*C. arimucosum* *C. diphteriae* *C. efficiens* *C. genitalium* *C. glutamicum* *C. halotolerans* *C. jeikeium* *C. kroppenstedtii* *C. pseudotuberculosis* *C. resistens* *C. timonense* *C. ulcerans* *C. urealyticum* *C. variabile*	2.82 2.48 3.22 2.35 3.31 3.22 2.48 2.45 2.32 2.60 2.55 2.56 2.36 3.43	60.5 53.5 62.9 62.7 53.9 68.3 61.4 57.5 52.2 57.1 66.7 53.4 64.2 67.1	2,630 2,392 3,064 2,290 3,122 2,930 2,181 2,083 2,187 2,230 2,456 2,355 2,045 3,131

### Prophage genome properties

Prophage Finder [41] and PHAST [42] were used to identify potential proviruses in *C. timonense* strain 5401744^T^ genome. The bacteria contains at least one genetic element of around 40.3 kb (with a GC content of 64.9%), we named CT1, on contigs 6-7. A total of 53 open reading frames (ORFs) were recovered from CT1, that were longer than 55 amino acids and most of them (44) encode proteins sharing a high identity with proteins found in *Actinomycetales* order viruses. The preliminary annotation of CT1 was performed and the majority of the putative genes (41) encode hypothetical proteins. The ORFs with an attributed function (12) encode proteins involved in DNA packaging, cell lysis, tail structural components and assembly, head structural components and assembly, lysogeny control, DNA replication, recombination and modification. 47 of the ORFs are located on one strand and 6 on the opposite strand.
